# On the Varieties of Cataract; Their Causes, Formation, and Cure

**Published:** 1828-02

**Authors:** S. J. Stratford

**Affiliations:** Assistant Surgeon 72d Highlanders.


### THE LONDON
### Medical and Physical Journal.

N° 348, VOL LIX.] FEBRUARY, 1828. [N° 20, *New Series.*

For many fortunate discoveries in medicine, and for the detection of numerous errors, the world is indebted to the rapid circulation of Monthly Journals; and there never existed any work, to which the Faculty, in Europe and America, were under deeper obligations, than to the Medical and Physical Journal of London, now forming a long, but an invaluable, series.—RUSH.

### ORIGINAL PAPERS,
AND
### CASES OBTAINED FROM PUBLIC INSTITUTIONS AND OTHER AUTHENTIC SOURCES.

### CATARACT.

*On the Varieties of Cataract; their Causes, Formation, and Cure.*

By S. J. Stratford, Assistant Surgeon 72d Highlanders.

A great difference of opinion still existing in the minds of the profession respecting the causes and formation of cataract, I have been led, from a variety of circumstances, to pay particular attention to this disease; so that I trust the following observations may be deemed worthy of consideration: they may appear novel, but I hope they will be fully supported by future observation and experience.

To comprehend the nature of cataract, we must have a correct idea of the anatomical structure and formation of the parts concerned: we must attend particularly to the connexion between the lens and its capsule, and have a just conception of the minute, delicate, and transparent tissue by which the lens is held in connexion with life and the animal economy; and, if we pay due attention to its natural and healthy condition, we shall be better able to comprehend and appreciate the varieties of its diseases. I shall, therefore, pass briefly in review the anatomical structure of the lens and its capsule, conceiving that it will the better lead us to understand this disease.

The crystalline lens is that transparent body placed upon the anterior surface of the vitreous humor, imbedded as it were in its substance; it is covered and surrounded by a beautifully transparent capsule, which serves to retain it in its proper situation; we observe that the lens is doubly convex, and that in its posterior surface this is much more marked than in its anterior. The lens is formed by very thin concentric lamellæ, superposed upon each other, increasing in density, and finally surrounding a harder nucleus. The more external layers are soft, and easily removed, but the nearer we approach the centre these become more condensed and firm in their texture: as a necessary consequence of this formation, we find the centre considerably thicker than the circumference. The component parts of the lens, chemically examined, seem to consist principally of albumen and gelatine, with traces of several neutral salts; it is firmly coagulated by boiling, or the action of acids. A fact curious in the formation of the lens is, that by the action of heat it is separated into thin equal divisions: this is the separation of its fibrous texture, and no proof of its muscularity, as was imagined by the ingenious Dr. Younge. The lens is contained in its proper capsule, a thin transparent membrane, which is firmly connected to the surrounding textures. This capsule is described by some anatomists to be formed by a splitting of the external membrane of the vitreous humor; but this would form a lamina so very thin, that it would not be strong enough to confine the lens in its situation, but would be ruptured by every motion of the globe. Moreover, if we compare the relative density of this membrane with the capsule, we shall find the capsule considerably thicker than the membrane, especially when it approaches the lens, which must at once convince us of the fallacy of such a conclusion. The capsule undoubtedly is a distinct membrane, firmly connected, and surrounded by the adjoining textures. It is strengthened and supported on the fore part by a reflection of the tunica Jacobi, which, having surrounded the proper matter of the nerve, is continued forward, comes in apposition with, and is connected to, the posterior surface of the ciliary processes and external layer of the hyaloid membrane. At a short distance, however, from the capsule of the lens, this membrane separates from the vitreous humor, and, proceeding forwards, is reflected over the capsule of the lens, firmly adhering to it, and serving to keep it in its proper situation; while the outer layer of the hyaloid membrane is situated behind, and, being closely united to it, assists to fix it in its position. Between the adhesion of the tunica Jacobi to the hyaloid membrane and its reflection over the capsule of the lens, we find a cavity denominated the canal of Petit, or, by the French, canal Godronné; this being regularly crossed by little transparent fibres (in all probability, serous arteries and veins,) passing from the union of these membranes to the capsule of the lens, serving to bind down the former tissue at certain intervals; so that, if we open into this cavity, and inflate it by means of a blowpipe, the air passes easily around the capsule of the lens, and elevates the tunica Jacobi at equal distances, forming a kind of crown, having the lens contained in its capsule for the centre. Within the capsule we find a minute quantity of fluid, which surrounds the lens, and is called the liquor Morgagni, which in all probability is to fill out the capsule, and permit the motion of the lens in the adjustment of the eye to near and distant vision: this fluid must be secreted by the inner surface of the capsule.

It has been said that the lens is not supplied with blood-vessels; that it is only a deposition from the liquor Morgagni by which it is surrounded. That this is not the fact, I am convinced; and I am sure I shall be supported by every rational and thinking mind; for, as the lens is formed by an infinite wisdom for a particular and useful purpose, so must it be supplied with vessels which nourish and connect it with the general system, supporting it in that combination of actions which is a proof of its vitality, and even of its existence as a part of the animal frame. As a positive proof, in the healthy eye we find it perfectly transparent; but, when separated from its connexions, it soon becomes opaque, and is then absorbed: this must convince us that it possesses life, and therefore has vessels circulating through it, by which this life is supported. These vessels pass, I believe, (although I cannot positively demonstrate the fact,) from the capsule to the circumference of the lens, taking their course through the liquor Morgagni, and carrying a perfectly transparent fluid: so minute indeed are they, that they may be ruptured by a blow on the eye, when they leave the lens as a foreign body, similar to what anatomists would have us to believe was its natural condition. The vessels which supply the capsule of the lens I have often seen ramifying on its surface in the living eye during disease: in their natural condition, however, they are perfectly invisible, being minute serous twigs from the arteria centralis retinæ. The arteria centralis retinæ, having supplied the retina and arrived at the ciliary processes, inosculates freely with the short ciliary arteries, and going forward as serous vessels through the canal of Petit, and by their adhesions causing the roseated appearance already mentioned, they give out minute serous twigs to the membranes forming this canal, while their chief branches go on to supply the capsule of the lens, and even the lens itself: besides these, the twig that passes through the centre of the vitreous humor is finally distributed on that part of the hyaloid membrane which strengthens and supports the posterior part of the capsule of the lens.

In this delicate system of vessels, there must be some which act the part of arteries, conveying and depositing the materials which compose the lens; while again there must be others, which absorb and remove that which has become effete and useless; for it is under this tenure only that the lens can possess life, and form a part of the animal frame.

The uses of the human lens would appear to be similar to the optician's, but it is much more perfect in its construction: it is evidently placed behind the pupil to refract and concentrate the rays of light, so that they impinge with due precision upon the sensible retina, the more essential portion of the organ of vision.

Having thus spoken of the formation of the lens and its capsule, I come to the consideration of the varieties of cataract, which essentially consist in an opacity of the lens, its anterior or posterior capsule, either of which may be separately affected, or all be combined in the same eye. When they are obvious to our sight, they are marked by distinct appearances that seldom or never fail us in our diagnosis.

As a general rule, we may say that an opacity of the anterior capsule is known by its dull white appearance, which something resembles common tissue paper;—an opacity of the lens only is marked by its shining surface, which is perceived to be the thin transparent capsule still covering the opacity;—and when the posterior capsule only is affected, it takes on a dirty white appearance, and by a practised eye is soon distinguished, from its evident depth of situation. These means of diagnosis will generally serve us, but they will be met with under a great variety of different circumstances, which must be minutely known to enable us to form a correct distinction of the situation of the disease.

The immediate causes of the formation of cataract have long been involved in much obscurity, but, by carefully tracing each variety of the disease from its source, and comparing the several causes and effects, I trust I shall be able to point out on what cause each may depend, which will greatly simplify the description, at the same time that it points out the kind of operation which ought to be used in the cure of these different varieties.

Inflammation is undoubtedly one of the causes of cataract: it may be either acute or chronic, and, with respect to its effect, either complete or partial. When cataract is a consequence of the acute variety, it is always complete; that is, both the lens and capsule have participated in the disease, and have become wholly opaque. This opacity is caused by a deposition of coagulable lymph within their previously transparent tissues: with this we frequently find a closed and permanently contracted pupil, for the most part adhering to the capsule of the lens. This condition of the parts prevents our seeing the peculiarities of the disease; but when the iris is not implicated, or but very partially, in the complaint, we shall observe a dense opaque capsule, which sometimes contains a somewhat enlarged lens. The opaque capsule has the dull white appearance noticed above, which particularly distinguishes it from a lenticular opacity; while, if the lens is enlarged, or there is a greater secretion of the liquor Morgagni, the posterior chamber of the aqueous humor is abolished, the iris sluggish in its actions, and presenting a black mark around its pupillary margin, which is most distinctly seen in a blue eye: this is formed by an eversion of the uvea, or pigment, on the posterior surface of the iris, pushed forward by the increased size of the lens. Sometimes even the iris is pushed out of its position by an enlarged and protruding lens; so that, if the pupil was previously contracted, upon the first application of the belladonna, it becomes largely and permanently dilated, provided it has formed no previous adhesions. The lens has lost its transparency, but we cannot observe it through the morbidly thick and opaque capsule; but, when we come to operate upon it, we shall find that it is not much firmer or harder than the natural structure; a circumstance of very material consideration in the choice of our operation. If any adhesion has taken place between the uvea and capsule of the lens, the application of the belladonna may rupture their connexions, and leave a considerable portion of the pigment adhering to the capsule: this forms a variety of the complaint that has been denominated the Cataracta Choroidalis. The acute progress of the disease is marked by all the symptoms of internal inflammation, such as considerable vascularity of the sclerotic coat, pain in the eye and temple, while generally more or less affection of the iris is present. The effusion of lymph into the transparent tissues is so sudden, that we are seldom able to mark the progress of the disease but by its ultimate effect.

The inflammatory action may be chronic, when the opacity will be more or less complete, according to the intensity or duration of the disease. This morbid action, which I have ventured to designate by the term Chronic Inflammation, generally takes its course slowly, without much pain, so that in some cases it does not rouse the attention or exert the fears of our patient. Sometimes, however, very soon after the commencement of the disease, he finds that objects appear indistinct, and, if examined with attention, they seem covered with a mist, or as though he looked through a glass that had been breathed upon; while, at night, the candle appears to have a halo or brim around its flame. These, indeed, are some of the most distinctive marks of incipient opacity in some of the transparent media: they cannot possibly be confounded with disease or insensibility of the optic nerve; for this always presents to the imagination a dark spot, or general deficiency in the power of vision. If we now apply the belladonna so as to dilate the pupil, (which should be an invariable practice, so that we may observe all the peculiarities of the complaint before we pretend to form our judgment of its kind,) the patient will be better able to distinguish objects, from the dilated pupil admitting more of the rays of light to fall upon the retina. The same effect occurs in the evening or a dark day, which is a symptom that would strongly confirm our suspicions of the nature of the disease, even before we recognise an opacity; while, if the disease is in the nervous texture, sight is best in a good light. These symptoms, I imagine, rather indicate an increase in the density of the fluid circulating in the transparent media; but, should the morbid action go on to the deposition of lymph, it soon becomes visible, upon examination of the pupil. Should the disease subside or be diminished by proper remedies, the lymph may again be absorbed; but, should it become organised, the opacity will be permanent. As the disease proceeds, a number of spots, or opacities, will be observed in the pupil, at regular distances, around the circumference. Sometimes we may perceive two sets,—one in the anterior capsule, more white and distinct; the second of a yellowish muddy colour, marking, by the obvious depth of its situation, that its seat is in the posterior capsule. At this time, too, we may frequently see several triangular-shaped stripes, that often take on an appearance like mother-of-pearl, or, if more opaque, look like spermaceti when broken across; and, should these striæ become completely opaque, they form the striated Capsular Cataract. These striæ almost always pass from the circumference to the centre; which peculiarity may, perhaps, be accounted for by the manner in which the vessels pass to the supply of the part. When the disease has arrived at this point, it may remain stationary for years; or, if it go on, it may become complete, the capsule being entirely opaque, as marked by its dull white colour. Before the disease, however, has arrived at this extent, we may observe the lens to put on a bluish white colour, which is more marked in the centre than the circumference, while the partially opaque capsule is evidently seen stretched over it: this by degrees becomes more opaque, but it is generally soon hid from our observation by the dense opacity of the capsule. If at this period we cut up the lens, we shall generally find it of its natural consistency, though in some few instances it may have become harder and rather firm, which, when in situ, is marked by its assuming a darker tint, deeper situated than the capsular opacity; and, if it should become enlarged, it will encroach upon the posterior chamber of the aqueous humor.

I have noticed that the opacity may be partial and remain stationary in the anterior capsule; the same may occur in the posterior, which, as I remarked, is particularly known by its yellowish muddy-white appearance, more or less dense according to the intensity of the disease. It may commence either in the centre or at the circumference, when it takes the form of striæ, which, if carefully examined, may be observed to have a concave shape, evidently (to an eye practised in the observation of these diseases) deeper seated than the lens.

It will, perhaps, be correct here to notice that I have, in several instances, observed opacities evidently deeper seated than the last variety of these diseases: these, I think, may be correctly referred to an opacity in the vitreous humor, which might mislead an inexperienced person with respect to their immediate seat.

Besides the symptoms already mentioned as attending chronic inflammation of the lens and capsule, we shall generally find that there is a greater determination of blood to the eye than usual; and this is especially evident after an examination of the organ. Many distended veins will be seen ramifying in the conjunctiva, while a slight pink-coloured halo surrounds the margin of the cornea; and often the eye is much more irritable than usual.

There is another variety of morbid action producing cataract, much more difficult to understand than the preceding: this always commences in the lens itself, is never observed but in old people, and would seem to be the product of a chronic action bearing some analogy, but evidently differing from the preceding varieties. It is always slow in its progress; so that, when the opacity first commences, the patient experiences the same indistinctness of vision as is mentioned in the foregoing variety: he has the same cloud before the eye, and observes a similar halo around the flame of the candle. The iris commonly acts with freedom, but the pupil is generally dilated, from the diminution of the number of the rays of light that pass through the only partially transparent lens. If we examine the dilated pupil, we soon observe that the lens is of a greyish amber or yellowish colour, more dark at the centre than at the circumference, which obviously depends upon the varied thickness of the body; and, if the lens only is affected, it has a fine polished surface. As the disease increases, the patient becomes more or less blind, and the lens assumes a darker colour, verging from yellow to a natural brown. Soon after this, we generally observe that the capsule begins to participate in the disease: at first it is but very slightly affected; the lymph is deposited in its texture, it takes on a dotted or variegated appearance, which has sometimes been compared to the white veins of some kinds of marble, and from this circumstance it has been denominated the Cataracta Marmorata. As the affection increases, so the capsule becomes more opaque, and in time perfectly obscures the hard lens, so that at this period we may be deceived in the nature and consistency of the cataract. The only points that will assist our diagnosis is the age of our patient, and the commencement of a similar disease in one of his eyes; for this complaint is not often confined to one eye, although it is seldom equally forward in both. The lens, we find upon an operation, is seldom larger than natural, but it is always observed to be considerably harder: this varies with the depth of its colour, which forms an excellent criterion of its consistency. When we examine it after the operation of extraction, we find that its centre nucleus bears a relative proportion to the general texture of the lens, so that, while the circumference is hard and firm, this has in some instances been said to equal bone in the density of its texture. The capsule, too, in the latter stage of the disease, is always much more tough than natural, is perfectly white in its colour, and opaque in its consistence, so that it effectually obscures the hard lens.

There is still another variety of cataract, obviously differing from any of the preceding; it occurs in all ages, while it is most frequently seen in the infant's eye, and is commonly known under the appellation of Congenital Cataract. In the commencement of this disease, (if we are so fortunate as to meet with a case,) we may observe that the lens at first presents a bluish white opacity, still covered by its shining capsule. If we watch its progress, we find the lens gradually becomes perfectly opaque and quite white, but from its colour is obviously soft; after a time its capsule is dotted with opacities, or we may observe streaks upon its surface of the dull white colour before mentioned: these increasing, the opacity becomes general, and now we frequently observe that it contains a fluid, which is of different degrees of density; so that, by attentive observation in the pupil largely dilated with belladonna, we can see, if the eye be kept quiet for a time, that the more white and dense particles have subsided, and now occupy the lower margin of the pupil; but, upon the first motion of the ball, they are again mixed in the general mass, and the cataract assumes an equible appearance. This is the Milky Cataract of some authors. At this period the capsule is commonly swelled out, as may be known by its effects upon the iris: this generally continues for a short time, when its size again begins to diminish, and that appearance of the iris to subside; while soon it will be seen to become smaller than before, and we may sometimes observe a dark ring around its circumference, evidently not produced by the everted margin of the iris, but depending upon the collapsed state of the capsule, permitting the rays of light to pass between it and the ciliary processes, and bringing to view the dark choroid coat through the transparent vitreous humor. The iris now may sometimes appear to have a vacillating motion, dependent upon the loss of support which the containing membrane of the eyeball experiences upon the removal of the lens from its natural situation; for at this period the lens is almost entirely absorbed, so that it leaves but a thin scale, or perhaps but its original centre nucleus: even this, however, in time is removed, and the anterior lamina of the capsule retires upon the posterior, until approximated they seem to form but one membrane, perfectly opaque, white, and elastic: the Cataracta Arida Siliquosa of authors.

Nobody, having just ideas of the anatomy, can contemplate the progress of this disease without at once recognising cause and effect; and if, in the congenital variety, any doubt should still exist, we have but to reflect upon the cause and termination of that cataract, sometimes produced by a blow upon the eyeball, or a rupture of the capsule permitting the escape of the lens, to be convinced of the fact. Indeed, if we examine the eye after death, and observe with what facility the lens starts from its capsule, we may perhaps be able duly to appreciate the tensity of those vessels that form its connexion with the capsule; and then, if we see how easily the lens escapes, we must at once perceive how slight must be that concussion which will break through these vessels, and how they may be broken without the capsule being opened. Should the lens then be separated from the living body, it is dead; becomes a source of irritation, though still enveloped in its capsule, and in process of time will be absorbed. I think I may say, then, that this variety of cataract always arises from a blow upon the eyeball, a sudden concussion, or unusual pressure, that acts upon the part so as to rupture the vessels that support the life of the lens; for, when this occurs, the lens gradually becomes opaque, and, as a foreign body, produces a degree of irritation upon the inner surface of the capsule, and lymph is deposited; while an increased secretion of the liquor Morgagni also takes place. The vessels that deposit the lymph upon the capsule may sometimes be seen by the naked eye: at first it is laid down but partially, and only at certain points, which causes a spotted appearance. The lens now begins to dissolve in the liquor Morgagni, and is slowly absorbed with that fluid. Soon after this the capsule becomes wholly opaque, but still it is evidently swelled out by an increased secretion of the liquor Morgagni: this projection, however, decreases as that fluid, and the lens dissolved in it, is absorbed; and, when it is entirely removed, and the laminæ of the capsule have collapsed upon each other, (in the infant eye,) we may perceive the black rim around its circumference. This, I believe, is caused by the capsule not increasing in size (now the lens is removed) in proportion with the rest of the eye, so that it remains stationary, while the vitreous humor and ciliary processes are fully developed.

From observation, I am led to believe that this is the most frequent cause of congenital cataract; the Cataracta Centralis, as recorded by authors, may sometimes occur; but this will depend upon partial inflammation of the capsule. I have observed the common variety of congenital cataract to follow tedious labours, in which the frontal bones, at this period of life separated into two portions, may experience a compression; they may even collapse over each other; when the pressure must affect the globe, and may rupture the vessels of the lens.

On referring to M. Demour's work upon the Diseases of the Eye, I am happy to find that I am supported by so excellent an authority; for he precisely states, that "Si on veut reflechir attentivement à la structure du cristallin, à son esolement dans sa capsule, à la petite quantité de lymph, ou humeur de Morgagni, qui l'environne, et a la nature de son envellope, on expliquera aisément les phénomènes que présente cette maladie pendent et après sa formation; tout medecin qui reflechira attentivement à la structure du globe de l'œil, trouvera, quelque soit alors le systême adopté pour expliquer la nutrition du cristallin, que *l'interruption de cette nutrition est la cause immediate de la cataracte.*

"Les causes externes de la cataracte sont connues: les blessures qui entament la capsule du cristallin; les *percussions qui detruisent la circulation dans ces parties si delicates; en un mot, tout ce qui peut changer les rapport du cristallin dans le globe detruisent sa transparence.*"

That this proceeding is the common process of congenital cataract, is also strongly corroborated by the opinions and observations of Saunders and Gibson; while their discrepancy of opinion may now be easily explained, by considering that they must have seen their patients at various periods, when different and distinct stages were present.

The cataract produced by a wound of the eye and a rupture of the capsule, with an escape of the lens, differs but little from that just noticed: it undergoes similar stages; the lens first becomes opaque, which commences immediately after it is separated from the capsule, and in a few days it is quite white; the capsule also is inflamed, and lymph is deposited on it; it becomes white, and in the end assumes the character of the coriaceous cataract.

By way of recapitulation, then, I think I shall be supported in considering that cataract may be caused—

1st. By acute inflammation of the lens and capsule.

2ndly. By chronic inflammation, for the most part beginning in the capsule of the lens.

3dly. A morbid action commencing in the lens itself.

4thly. By a rupture of the vessels which connect the lens with the capsule, and endow it with the principle of life.

5thly. By a rupture of the capsule, and escape of the lens.

These varieties of disease will obviously require different operations for their cure. Cataract caused by acute inflammation of the lens and capsule should always be cut up with the needle, and left to the process of absorption for its removal. That produced by chronic inflammation should experience a similar treatment. The third variety, in its very commencement, should undergo the operation of reclination or depression; but in its latter stage, when it has become hard, even should it be enveloped in a tough capsule, ought to be extracted; for the hard lens almost invariably causes so much irritation, that glaucoma is a consequence of its presence in the eyeball after it is depressed. The fourth or fifth varieties, by their progress, point out that we have but to separate the diseased portions from the living system by means of the needle, when the absorbents will entirely remove them from the organ of vision, and hereby cure the disease.

